# Y‐chromosome variability and genetic history of Commons from Northern Italy

**DOI:** 10.1002/ajpa.24302

**Published:** 2021-05-10

**Authors:** Stefania Sarno, Rajiv Boscolo Agostini, Sara De Fanti, Gianmarco Ferri, Silvia Ghirotto, Giorgia Modenini, Davide Pettener, Alessio Boattini

**Affiliations:** ^1^ Department of Biological, Geological and Environmental Sciences University of Bologna Bologna Italy; ^2^ Department of Life Sciences and Biotechnology University of Ferrara Ferrara Italy; ^3^ Interdepartmental Centre Alma Mater Research Institute on Global Challenges and Climate Change University of Bologna Bologna Italy; ^4^ Department of Diagnostic and Clinical Medicine and Public Health University of Modena and Reggio Emilia Modena Italy

**Keywords:** ABC modeling, demographic history, social‐cultural isolates, Y‐chromosome

## Abstract

**Objectives:**

Genetic drift and admixture are driving forces in human evolution, but their concerted impact to population evolution in historical times and at a micro‐geographic scale is poorly assessed. In this study we test a demographic model encompassing both admixture and drift to the case of social‐cultural isolates such as the so‐called “Commons.”

**Materials and methods:**

Commons are peculiar institutions of medieval origins whose key feature is the tight relationship between population and territory, mediated by the collective property of shared resources. Here, we analyze the Y‐chromosomal genetic structure of four Commons (for a total of 366 samples) from the Central and Eastern Padana plain in Northern Italy.

**Results:**

Our results reveal that all these groups exhibit patterns of significant diversity reduction, peripheral/outlier position within the Italian/European genetic space and high frequency of Common‐specific haplogroups. By explicitly testing different drift‐admixture models, we show that a drift‐only model is more probable for Central Padana Commons, while additional admixture (~20%) from external population around the same time of their foundation cannot be excluded for the Eastern ones.

**Discussion:**

Building on these results, we suggest central Middle Ages as the most probable age of foundation for three of the considered Commons, the remaining one pointing to late antiquity. We conclude that an admixture‐drift model is particularly useful for interpreting the genetic structure and recent demographic history of small‐scale populations in which social‐cultural features play a significant role.

## 1 INTRODUCTION

Among the evolutionary forces shaping the genetic variability of modern human populations, drift and admixture are of the uttermost importance. In general, both of them can act at the same time on a given population, albeit with shifts from one to another depending on the historic/demographic vicissitudes experienced by different human groups. In particular, genetic drift is usually associated with isolated populations, that is groups with a moderate demographic size and low exchange with other populations. However, migration and admixture can play a significant role even in isolates, thus contributing to the peculiar genetic structure that these populations frequently exhibit. Accordingly, in this study with ‘drift’ we mean the effects of regional/local isolation, while ‘admixture’ more precisely refers to long‐range migration events. Well‐known examples of this combination are ethnic‐linguistic minorities such as the thoroughly investigated Arbereshe and Greek‐speaking groups from Southern Italy (Boattini et al., 2011; Destro Bisol et al., 2008; Sarno et al., 2016; Sarno et al., 2017; Sarno et al., 2021; Sineo et al., 2014). The genomic history of these populations may be summarized according to the following scheme: (a) a founding migration event; (b) admixture—to a lesser or higher extent—with local groups; (c) isolation and drift, usually caused by a combination of cultural and geographical factors. Paradoxically, isolation and drift contributed to the conservation, in these groups, of clear genomic traces of their migration history. Furthermore, it has been shown that these populations may function as privileged observatories for detecting and reconstructing specific demographic events, whose genetic traces were diluted or disappeared in more general contexts (Boattini et al., 2015; Gokcumen et al., 2011; Sarno et al., 2016).

Ethnic‐linguistic minorities—where the exogenous origin of (at least part of) the group is usually well‐documented—are not the only cases to which an admixture‐drift model could be applied at a micro‐geographic scale. In this study, we investigate the genetic variability of a set of populations which share some aspects with isolates but whose origin is less clear or not known at all. For reasons of simplicity, we will refer to these populations with the term of “Commons,” which actually designs a peculiar way of sharing and devolving collective resources. According to the Digital Library of the Commons (http://dlc.dlib.indiana.edu/dlc/), these are juridical institutions based on “shared resources in which each stakeholder has an equal interest.” Italy hosts a wide archipelago of Commons, each of them with its own features and history, which makes them a particularly interesting set of populations to explore. Our first research in that vein regarded the *Partecipanza* of S. Giovanni in Persiceto, a Common located in the Padana Plain whose members form the patrilineal descent of a group of well‐identified founder families. Indeed, that study suggested that: (a) an admixture event, probably involving a group of Germanic origins, was at the origin of the Common and (b) subsequent isolation helped to preserve trace of such event in the Y‐chromosomal structure of the Partecipanza (Boattini et al., 2015).

**FIGURE 1:**
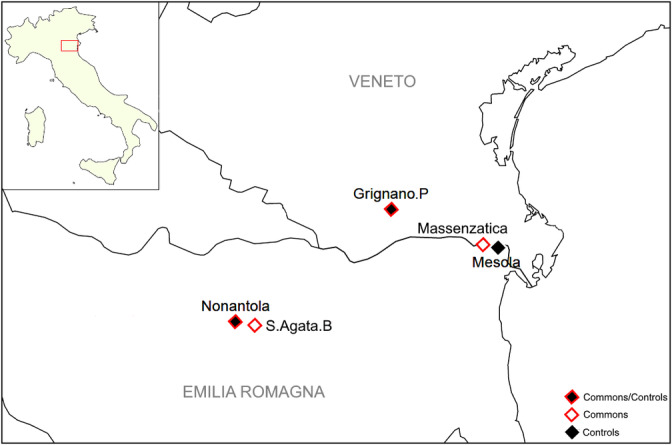
Geographic map showing the location of the analyzed Commons and Controls

Here we focus on four more Commons from the Padana Plain in Northern Italy (Figure 1). Two of them (Nonantola and S. Agata Bolognese) are located in the Central Padana Plain (from now on Central Commons), in the same area of the previously mentioned S. Giovanni in Persiceto, with which they also share the denomination of *Partecipanza*. The remaining two (Grignano Polesine and Massenzatica) are at the Easternmost fringes of the Padana Plain, not far from the Po delta (from now on Eastern Commons).

All these Commons share a number of features, most notably all of them evolved from typical medieval contracts called emphyteutic leases, according to which the holder had the perpetual right to the enjoyment of a given property in exchange of some conditions, most notably the increase of the value of the land (*ad meliorandum* clause), the payment of a small rent and the obligation of stable residence. In our case, emphyteutic concessions were established between important abbeys (Nonantola for Central Commons and Pomposa for Eastern Commons) with vast but deserted properties and local communities as a whole. In exchange of that, the locals contributed significantly to land reclamation and peopling of the territory. At a later time most of these Commons “closed” their ranks and reserved the right to share the collective lands to a restricted group of “founder” families (Arioti et al., 1990; Costato, 1968; Cori, 2011; Fregni et al., 1992). Indeed, three of the four Commons considered here (namely Nonantola, S. Agata B., and Grignano P.) still limit the access to the shared resources to the legitimate, paternal descent of a given set of families/surnames. In addition, and in all the cases, stakeholders must maintain their place of residence within the legal boundaries of the Common. The combined effect of these rules is a long‐term relationship between population and land, modeled upon the shared property of the Common.

With this study, and in consideration of the patrilineal nature of most of these Commons, we specifically explore their paternal (Y‐chromosome) genetic heritage with the aim of detecting effects of the peculiar social‐cultural features related to their presence in the corresponding populations. In particular, we will (a) evaluate if a drift‐only model can fully explain their genetic structure or if a model encompassing both admixture and drift provides a better fit and (b) reconstruct their genetic histories and their implication for the wider scenario of Northern Italy.

## 2 MATERIALS AND METHODS

### 2.1 The populations

Among the considered Commons (Table 1, Figure 1), all Central Commons are *Partecipanze*. Currently, six *Partecipanze* are still present and active in the study area: Nonantola, S. Agata Bolognese, San Giovanni in Persiceto, Cento, Pieve di Cento, and Villa Fontana. Among them, the here studied Nonantola and S. Agata Bolognese occupy the westernmost position. Their assets extend for 765 and 553 ha, respectively, while the number of stakeholders is 2814 (2009) and 237 (2011) (Arioti et al., 1990; Fregni, 1992; http://www.partecipanzanonantola.it; https://www.partecipanza.org/). The history of these Commons is quite similar, though the case of Nonantola is best documented. Traditionally, the origins of the Partecipanza of Nonantola are traced back to 1058, when emphyteutic grants were stipulated between the local abbey and the community, but only in 1584 the Common closed its ranks to external families (“*chiusura dei ruoli*”). This was only the beginning of a bitter fight between two factions, one of them formed by peasants (“*bocca viva*”), and the other by rich landowners, many of them foreigners (“*bocca morta*”). Such issue was settled only in 1915, when the descendants of the peasants liquidated the landowners group. In the meantime, in 1856 the Partecipanza separated its administration from that of the municipality and in 1961 finally severed its 1000‐year old ties with the Abbey of Nonantola (Venturoli, 2004). S. Agata B. had a less complicated history, being able of redeeming its assets from the Abbey already in 1577 and separating from the municipality in 1797 (Arioti et al., 1990; Fregni, 1992). Both these Commons, as all other *Partecipanze*, are characterized by a peculiar procedure called *cavazione*, which is a periodic re‐shuffling of shared lands among heads of the participating households. Currently in Nonantola this happens every 18 years and in S. Agata Bolognese every 9 years (Arioti et al., 1990).

**TABLE 1:** Sample information and details about the considered Commons

Location	Type	Criteria	Area (ha)	N. Stakeholders	N. Samples	
					Common	Control
Nonantola	Partecipanza	Pat, Res	765	2814 (2009)	54	55
S. Agata Bolognese	Partecipanza	Pat, Res	553	237 (2011)	49	
Grignano Polesine	Comune	Pat, Res	130	327 (2012)	66	49
Massenzatica	Formerly Comune, now Consorzio	Res	353	640 (2020)	51	42

*Note:* Pat: legitimate patrilineal descent of a given set of founder families/surnames. Res: residence within the legal boundaries of the Common.

The Eastern Commons here considered are Grignano Polesine and Massenzatica. The Common of Grignano P. is currently named *Antichi Beni Originari* and it is referred to by locals as *Comune*. Its shared assets are extended for 130 ha and the number of stakeholders was 327 in 2012 (http://www.antichibenioriginari-grignano.it/). The organization and functioning of the *Comune* of Grignano P. are very similar to *Partecipanze*, including a periodic re‐shuffling of the shared lands that in this case is performed every 5 years. The *Comune* originated from an emphyteutic grant from the Abbey of Pomposa probably during the central middle ages. This Common closed its ranks to external families already at the end of 15th century (1494) and redeemed its assets from the Abbey in 1968 (Costato, 1968). As for Massenzatica, the current name of the Common is “*Consorzio Uomini di Massenzatica*.” The origin of this Common is usually associated to an 1182 emphyteutic grant from the abovementioned Abbey of Pomposa. Differently from the *Partecipanze* (Nonantola, S. Agata B.) and the *Comune* (Grignano P.), Massenzatica never established a closure of the ranks to foreigners/immigrants neither procedures of re‐shuffling among the stakeholders. The right to benefit of the shared goods is here allowed to all male heads of the household permanently residing in the villages of Massenzatica, Monticelli and Italba. The extension of these goods is 353 ha and the number of stakeholders is presently 640. The Common of Massenzatica reached its present form in 1894 with the creation of the “*Consorzio*” and the resolution of all the easements that, from the times of Pomposa, still weighed on the land (Cori, 2011; http://www.uominidimassenzatica.it/).

From the social‐cultural point of view, there were no significant differences between members of Commons and their neighbors: they spoke the same dialect, they shared the same religion and cultural identity. Furthermore, up to 19th century usually Commons were not separated from their municipalities, meaning that local civic administration coincided with that of the Common. Of course, this fact could entail some advantages for Common members, who in addition could count on the availability of unalienable land portions in an eminently agricultural society and on the collaboration/mutual assistance among themselves (Fregni, 1992).

As these advantages disappeared or became less important since the economy of the region shifted towards industry and tertiary, members of Commons however conserved the greatest regard for their ancient institutions and their traditions, which in turn had and still have a great impact in shaping the territory on which the community is settled. Starting from the late 18th century and especially after the Napoleonic period, Commons experienced long and bitter fights with public authorities, which manifested increasing hostility toward the peculiar way of possessing that these institutions embodied. As a result, some Commons disappeared during this period turning into “normal” private properties. It can be said that the still‐existing institutions are only the remnants of a wider phenomenon (Alfani & Rao, 2011; Mantovani, 2017).

Finally, Commons were (and are) not “closed” communities to the “outside world” also from the demographic point of view, as suggested by the relatively low number of endogamic marriages (see also results below), save the partial exception of Nonantola in which an increase of endogamy rates was indeed measured after the “*chiusura dei ruoli*” in 1584 (Alfani, 2015).

### 2.2 DNA samples

For each location, we sampled both individuals from the Common and individuals residing in the same place, hence sharing the same environmental and cultural features, but not belonging to the Common itself. We refer to these last samples as “Controls.” For the case of Massenzatica, Controls were sampled in the locality of Mesola, which is the administrative center of the municipality that includes the Common. The Partecipanze of Nonantola and S. Agata Bolognese, given their geographical proximity, share the same Control group, which was sampled in Nonantola. In total, we collected 366 samples, 220 of them belonging to Commons and 146 to Controls (Table 1).

All these samples were collected according to the standard “grandparents” criterion (i.e., at least three generations of ancestry in the area of the considered Common) and by excluding related individuals. As for the Control samples, all the selected individuals share surnames that were identified as autochthonous for the considered area by Boattini et al. (2012). Data from 63 individuals have been previously published in Boattini et al. (2019). The collection of biological samples was performed during various sessions from 2015 to 2017. For all subjects, a written informed consent was obtained and the Bioethic Committee of the University of Bologna (Italy) approved all procedures. The confidentiality of personal information for each participant to the study was assured and the research was performed in accordance with relevant guidelines and regulations for studies involving human subjects stated by the WMA Declaration of Helsinki.

Whole genome DNA was extracted from buccal swabs by using a salting out protocol modified from Miller (Miller et al., 1988) and quantified with the Qubit® dsDNA BR Assay Kit (Life Technologies).

### 2.3 Y‐chromosome genotyping

All samples were amplified for the 23 Y‐STRs loci included in the PowerPlex® Y23 System (Promega) following manufacturer recommended protocols. PCR products were sized on an ABI PRISM 310 Genetic Analyzer and alleles were called with GeneMapper ID software (Thermo Fisher Scientific) according to the manufacturer's instructions.

Next, all individuals were additionally genotyped for 42 Y‐SNP loci using multiplex SNaPshot mini‐sequencing assays (Thermo Fisher Scientific), as described in Sarno et al. (2014). The SNP genotyping was carried out by means of PCR Multiplex amplification, followed by Minisequencing reaction based on dideoxy Single Base Extension (SBE), which was performed with the SNaPshot multiplex kit (Applied Biosystems). SBE products were finally analyzed through capillary electrophoresis on an ABI Prism 310 Genetic Analyser.

Y‐STRs data for comparison populations were extracted from the literature for both Italy (Boattini et al., 2013) and Europe (Purps et al., 2014), respectively. In addition, comparison data for within‐haplogroup comparisons from 16 Euro‐Mediterranean populations (329 samples) were also considered and retrieved from Hallast et al. (2015).

### 2.4 Pedigree reconstruction

The *Partecipanze* (Nonantola and S. Agata B.) and the *Comune* (Grignano P.) kept records of all the households participating to the sharing of leased assets. These records were regularly updated in occasion of the abovementioned periodic re‐shuffling of the lands among the householders. Such detailed historic‐demographic information allowed the reconstruction of paternal pedigrees up to the late 16th century for Grignano P. and up to 19th century for the *Partecipanza* of S. Agata B. Unfortunately, records from the *Partecipanza* of Nonantola could not be accessed after the earthquake of 2012. When more than one individual was found to share a recent paternal ancestor, they were grouped into a single pedigree.

### 2.5 Statistical methods

#### 2.5.1 Generation time

We calculated average generation times based on reconstructed paternal pedigrees, using those individuals for which birth information was available. Rates were calculated by dividing the total number of years for the number of generations encompassed in all the considered pedigrees (after excluding the most remote ancestors, due to uncertainty of their birth date). Confidence intervals (95%) were obtained bootstrapping along different branches of pedigrees (1000 replications).

#### 2.5.2 Social endogamy

By inspecting all marriages included in reconstructed pedigrees (S. Agata B., Grignano P.), we considered as endogamic those marriages in which both partners bear surnames of the Common. Marriages for which the surname of the bride was not available were excluded from calculations. Final rates were calculated by dividing the number of socially endogamic marriages for the total number of marriages. Confidence intervals were computed based on a binomial distribution (where the size parameter is equal to the total number of marriages and the probability parameter is given by the observed endogamy rate) using the R function *qbinom* (R Core Team, 2017).

#### 2.5.3 Diversity indexes

Standard within‐population diversity parameters (Gene Diversity, Mean Number of Pairwise Differences, Nucleotide Diversity) for Y‐chromosome haplogroups and STR haplotypes were estimated with Arlequin software 3.5.1.2 (Excoffier et al., 2007). Comparisons of single haplogroup (hg) frequencies between Commons and Controls were performed with Fisher tests and p‐values were corrected using the Bonferroni criterion. The overall differentiation in haplogroup composition between Common‐Control pairs was assessed through AMOVA analysis based on pairwise Fst statistics as implemented in the above mentioned Arlequin software.

#### 2.5.4 Multivariate analyses between populations

In order to check the position of our populations within the Italian and European Y‐chromosomal genetic landscape, we performed a nonmetric multidimensional scaling (MDS). Because different studies used different levels of hg resolution, the analyses were based on the 23 Y‐STR haplotypes that were available for all the considered reference populations. Calculations were performed using the Rst genetic distances computed by the Arlequin software (Excoffier et al., 2007) and the function *isoMDS* implemented in the R software MASS package (R Core Team, 2017; Venables & Ripley, 2002). The first and the second dimension were represented in a scatterplot, along with the corresponding stress value.

#### 2.5.5 Within‐haplogroup comparisons

In order to explore the genetic variability within haplogroups exhibiting significant frequency differences between Commons and Controls, we used Discriminant Analysis of Principal Components (DAPC, Jombart et al., 2010) based on Y‐STR data as in Boattini et al. (2013, 2015). This analysis is aimed to (a) identify well‐resolved groups of haplotypes within haplogroups; (b) highlight possible affinities/similarities with reference haplotypes from Italy and Europe; and (c) constitute a starting point for time estimates. All the analyses were performed within the R software package *adegenet* (Jombart, 2008).

#### 2.5.6 Time estimates

Time estimates focused on the haplotype clusters identified by DAPC within the most frequent haplogroups and were limited to clusters including at least nine individuals and separate estimates were also performed for clusters with at least nine individuals within a specific Common. Y‐STR mutation rates adopted in the procedure were taken from Ballantyne et al. (2010). Because population events involving Commons are relatively recent, the biasing effect of Y‐STRs saturation through time is negligible (Boattini et al., 2016; Boattini et al., 2019) and all Y‐STRs (minus DYS385a/b) were therefore used for calculations. In addition, since estimates may be very sensitive to the presence of outliers, we adopted the outlier detection and exclusion procedure described in Boattini et al. (2013). Time estimates were calculated using two different approaches: (a) the *SD* estimator (Sengupta et al., 2006); (b) the Bayesian method implemented in the Batwing software (Wilson et al., 2003). As for the latter, we adopted a standard coalescent model with constant (effective) population size (N). Since we are considering not the entire population, but rather clusters of individuals within single lineages (as in Balaresque et al., 2010 and more recently Platt et al., 2017; Huang et al., 2018), we assigned a uniform distribution on the interval (10,1000) to the N prior. Accordingly, we set STR‐specific priors of mutation rates using gamma distributions in the form *gamma(nmut,ngen)*, where *nmut* is the number of observed mutations and *ngen* is the total number of meioses (as in Ballantyne et al., 2010). The number of times parameters were updated between samples (*Nbetsamp*) was 10, and the number of times trees were changed before updating parameters (*treebetN*) was 20. The number of samples between writing the outfile (*pigap*) was 1,500,000. A total of 3.5 million of MCMC runs with different random seeds were run for each haplotype cluster, and the first 1 million iterations were discarded as burn‐in. Time estimates were calculated from the resulting outfile using the product of the posteriors estimated for the population size N and the total height of the tree T. For both methods, a generation time of 33.72 years (as estimated by our analysis, see Results) was specifically used for converting all time estimates in years. All statistics were calculated with the R software.

#### 2.5.7 Model comparison by approximate Bayesian computation

**FIGURE 2:**
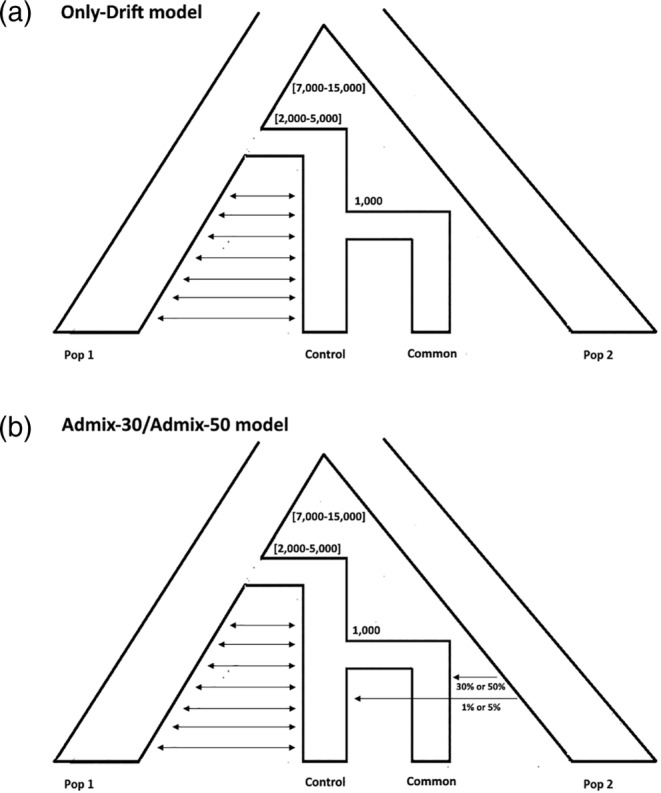
Demographic histories of the considered models. “Pop 1” represents a general Italian population; “Pop 2” represents a non‐Italian group, source of potential external admixture/introgression

In order to investigate the recent demographic history of the considered groups, we compared three different models for each Common/Control pair. All models follow the same demographic history (Figure 2) whose events are: (a) a divergence between 7000 and 15,000 years ago representing a putative separation between Italian and non‐Italian (Pop 2) groups; (c) a second divergence between 2000 and 5000 years ago representing the segregation of local populations (i.e., the ancestors of Common/Control pairs) from the general Italian population (Pop 1); (c) a third divergence event 1000 years ago representing the separation between Common and Control. In all models the local populations (from event 1 to event 2) and the Controls (after event 2) keep exchanging migrants with Pop 1 (general Italian population). The three tested models instead differ for the degree of admixture from non‐Italian Pop 2 (external source of genetic variation) to the Common. According to the “Only‐Drift model” (Figure 2a), there are no exchanges between these two demes, and the Common remains completely isolated until present times. Under the “Admix‐30 model,” 30% of the Common Y‐chromosomal composition and 1% of the corresponding Control composition originated from an external source of variation (Pop 2), whereas under the “Admix‐50 model” these percentages are 50% and 5%, respectively (Figure 2b). For a detailed description of all models' parameters see Table S1.

We generated 5000 simulated data sets (21 Y‐chromosome microsatellites) under each model with *fastsimcoal* (Excoffier et al., 2013) within the ABCToolbox suite (Wegmann et al., 2009). We sampled 40 individuals from the Common deme and 40 individuals from the corresponding Control deme at each iteration, while Pop 1 and Pop 2 were set as “ghost” populations. The considered summary statistics are: the number of alleles (K), Heterozygosity (H), Garza‐Williamson statistic (GW), allelic range (R), Fixation index (Fst), Pairwise differences (Pi), and mean delta‐mu squared (DMUSQR). We compared models using Random Forest Approximate Bayesian Computation (ABC‐RF: Pudlo et al., 2016). Under ABC‐RF, the Bayesian model selection is rephrased as a classification problem, in which the classifier is constructed from simulations via a machine learning RF algorithm. Once the classifier is constructed and applied to the observed data, the posterior probability of the resulting model can be approximated through another RF that regresses the selection error over the statistics used to summarize the data. To perform the model selection procedure, we used the function *abcrf* from the R package abcrf and employed a forest of 500 trees, as suggested by Pudlo et al. (2016). Before calculating the posterior probabilities of the most supported model, we computed the confusion matrix and evaluated the out‐of‐bag classification error.

In order to better define the patterns emerged from models' comparison, we performed the estimation of the admixture proportion in the considered Commons (introgression from Pop 2 to the Common). To do this, we designed a demographic model in which the admixture parameter is free to vary at each iteration from 0% to 50%. We performed 500,000 simulations and then estimated the posterior distribution through the *ABCestimator* tool of the ABCToolbox package (Wegmann et al., 2009).

## 3 RESULTS

### 3.1 Reconstructed pedigrees, generation time, social endogamy

In total, we reconstructed 61 paternal pedigrees (24 for S. Agata B. and 37 for Grignano P.; Figure S1, Table S2), encompassing 960 generations and 124 individuals. Of them, 28 refer to a single individual, 18 comprise two individuals and 14 more than two individuals (up to 6). Of these 124 individuals, 115 were actually genotyped while nine were excluded given their recent relatedness with other individuals, that is, a number of generations separating them from other individuals of the same paternal pedigree lower than 7. This was done for ethical reasons as in Claerhout et al. (2019) and Boattini et al. (2019).

Based on all 61 pedigrees, we estimated the average generation time and obtained 33.72 years per generation (95% CI: 33.22, 34.28). We then calculated values for each population, obtaining 34.18 (95% CI: 33.62, 34.74) for Grignano P. and 32.61 (95% CI: 31.64083, 33.58142) for S. Agata B. Hence, Grignano P. seems to exhibit slightly higher average generation times. Since Grignano P. pedigrees are averagely deeper than S. Agata B. ones, we also sub‐sampled pedigrees from the former in order to match the temporal depth of the latter. Results show that sub‐sampled pedigrees from Grignano P. yielded 33.91 years per generation (95% CI: 33.16, 34.77), which largely overlaps with the above estimate. This fact suggests that some, even if slight, population‐dependent variability indeed exists.

Social endogamy was evaluated for one of the central communities (S. Agata B.) and for one of the eastern communities (Grignano P.). Nonantola was excluded due to the current unavailability of historical‐demographical data and Massenzatica for not having a set of Common‐specific (“founder”) surnames. Grignano P. genealogies comprise 630 marriages, 569 of them including the surname of the bride. Social endogamy in these 569 marriages was 59.22% (95% CI: 55.01, 63.09). As for S. Agata B., our data set (pedigrees) comprises 335 marriages, 269 of them including the surname of the bride. Social endogamy here is much lower, being only 28.25% (95% CI: 22.68, 33.46). Therefore, these results show that, in these communities, patrilineal isolation was balanced by exogamous marriages.

### 3.2 Diversity indexes

**FIGURE 3:**
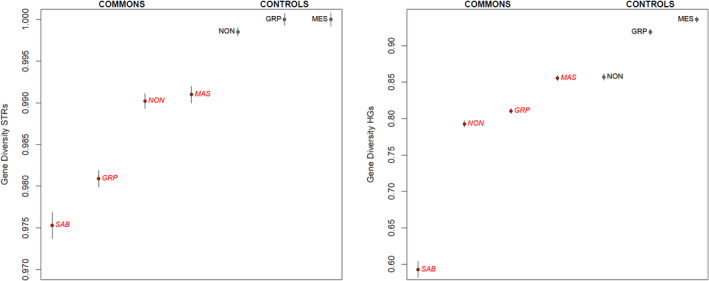
Y‐chromosome diversity indexes in Commons and Controls

We calculated classic indexes of genetic diversity for all the considered populations (Commons and Controls) using both Y‐STR haplotypes and haplogroup frequencies (Table S3, Figure 3). The obtained results (particularly those based on Y‐STRs) clearly show that all the considered Commons exhibit lower diversity values than the Controls. Interestingly this feature is observed also in Massenzatica, despite its higher social “openness” (membership is here conditioned only by residency) compared with the remaining Commons. As for these groups, the lowest diversity values were observed for S. Agata B., which in fact exhibits the lowest population size. Indeed, STR‐based Gene Diversity values for the three Commons based on founder families faithfully correspond to the number of stakeholders (Nonantola > Grignano > S. Agata B.).

When explicitly testing for genetic structuring among Commons, we obtain high and significant Fst values with both STR haplotypes and haplogroup frequencies (Fst = 0.042 with *p* value <0.001, Fst = 0.116 with *p* value <0.001, respectively). The same analysis with Control groups instead yields much lower Fst values, in one case (STRs) not even reaching the nominal significance threshold (STRs: Fst = 0.002 with *p* value = 0.330; haplogroups: Fst = 0.020 with *p* value = 0.006). These results confirm the strong differentiation among Commons, while Controls exhibit higher homogeneity between each other.

### 3.3 Haplogroup distribution

Haplogroup‐wise (Table S4), the considered Commons—despite generally harboring haplogroups typical of Western‐European populations—show some significant differences from their Controls, both from an overall point of view and for specific lineages. Let us consider first the two Central Commons, that is, Nonantola and S. Agata Bolognese. When performing overall Fst tests (AMOVA) based on haplogroup frequencies, both Commons appear as significantly different from the Control (Nonantola: Fst = 0.04, *p* value = 0.004; S. Agata B: Fst = 0.08; *p* value <0.001). In particular, these Commons, similarly to their Control (Nonantola), show a typical prevalence of R haplogroups, which make up to ~70% of their repertoire. However, Nonantola exhibits a significantly higher presence of R1b‐L2, when compared with the Control (33.33% vs. 7.27%, *p* value = 0.014), while S. Agata is strongly characterized by lineage R1b‐U152* (63.27% vs. 30.91%, *p* value = 0.0026).

Moving to Eastern Commons, again overall Fst tests revealed significant differences between them and their controls (Grignano P: Fst = 0.04, *p* value = 0.004; Massenzatica/Mesola: Fst = 0.03, *p* value = 0.005). As for specific lineages, Massenzatica, compared with its Control (Mesola), shows an overall higher frequency of R lineages (64% vs. 47.62%). Such difference is mainly due to a higher frequency of paragroup R1b‐L51*, however not reaching the statistical significance after Bonferroni correction (18% vs. 2.38%, *p* value = 0.386).

The case of the Common of Grignano P. is particularly interesting, since its main feature is the very high frequency (22.73%) of a non‐R haplogroup, T‐M70, which is absent in its control (*p* value = 0.002) and in general quite sparse in Italian populations (1.58%; Boattini et al., 2013).

### 3.4 Multivariate analyses

#### 3.4.1 MDS

**FIGURE 4:**
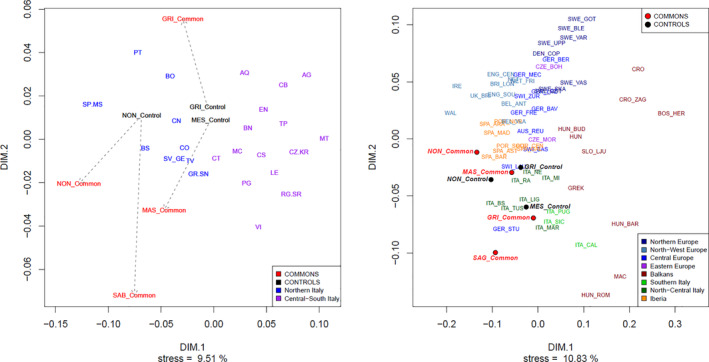
Nonmetric MDS representations of Commons. Controls and reference populations (Italy, *left*; Europe, *right*) based on Y‐STR data

In order to check the position of the considered groups within the Italian and the European Y‐chromosomal genetic landscape, we performed STR‐based MDS using several reference populations (Figure 4).

As for MDS results at an Italian level, we observe that Control groups are located towards the center of the plot, with Nonantola falling between North‐Western Italian populations and the two Easternmost Controls (Mesola and Grignano P.) mapped quite near to each other and in an intermediate position between North‐Western and South‐Eastern Italian population groups. Interestingly, all Commons occupy peripheral positions in the plot, which can be explained according to two hypotheses: (a) as a result of prolonged genetic drift (in agreement with diversity indexes results), and (b) as a result of admixture/introgression involving populations with a different genetic background.

When considering a European‐wide genetic space, the observed patterns are highly consistent with the previous case. In addition, the Common of Nonantola is placed along with Iberian populations, while the Common of S. Agata B. still occupies an outlier position.

#### 3.4.2 DAPC and time estimates

Within‐haplogroup analyses were performed for the most frequent haplogroups in the considered Commons, that is, R‐U152*, R‐L51, R‐L2, T‐M70 and G2a‐U8 (Table S5, Figure S2). DAPC results (Table 2, Table S5) show that these haplogroups revealed a number of well‐defined clusters variable from 3 to 7. In addition, all of them exhibit at least one Common‐specific cluster, that is, a cluster in which the majority (>50%) of the haplotypes is associated to a given Common. More precisely, S. Agata B. presents two associated clusters, both of them within R‐U152* (namely 4 and 5); Nonantola also presents two specific clusters, one in R‐U152* (Cluster 3) and one in R‐L2 (Cluster 4); Grignano P. exhibits six specific clusters in four different haplogroups, that is, R‐U152* (Cluster 1), R‐L2 (clusters 1 and 5), T‐M70 (Cluster 2) and G2a‐U8 (Clusters 1 and 3), but only four of them include at least nine individuals; Massenzatica (with Mesola) show one specific cluster within R‐L51 (Cluster 2), which is shared also with other Italian and non‐Italian populations. It is worth noticing that clusters from haplogroups R‐U152*, R‐L2, and G2a‐U8 instead did not show any match with non‐Italian comparison populations.

**TABLE 2:** Time estimates and standard errors (in years before present) based on DAPC clusters exceeding nine individuals

					Date_All		Date_Common	
Haplogroup	Cluster	Ntot	Common	Ncommon	SD estimator	Batwing	*SD* estimator	Batwing
R‐U152*	1	12	GP	12	‐	‐	1206.62 +/− 257.25	1280.524 +/− 141.75
R‐U152*	2	10	‐	‐	2541.43 +/− 541.83	3999.52 +/− 415.80	‐	‐
R‐U152*	3	19	NON	11	4714.93 +/− 1005.23	6996.91 +/− 575.771	1016.09 +/− 216.63	1535.75 +/− 206.22
R‐U152*	4	11	SAB	9	2674.84 +/− 570.28	2627.54 +/− 324.46	1040.1 +/− 221.75	1138.26 +/− 163.48
R‐U152*	5	15	SAB	15	‐	‐	520.59 +/− 110.99	594.17 +/− 76.30
R‐U152*	6	15	‐	‐	3139.15 +/− 669.27	4384.90 +/− 278.73	‐	‐
R‐U152*	7	11	‐	‐	3508.49 +/− 748.01	5832.41 +/− 491.53	‐	‐
R‐L51	1	7	‐	‐	‐	‐	‐	‐
R‐L51	2	16	MA/ME	10	2863.21 +/− 610.44	4457.97 +/− 377.79	1743.77 +/− 371.77	2276.28 +/− 311.76
R‐L51	3	17	‐	‐	2126.93 +/− 453.46	4814.91 +/− 321.75	‐	‐
R‐L2	1	7	GP	6	‐	‐	‐	‐
R‐L2	2	9	‐	‐	7405.99 +/− 1578.96	5023.37 +/− 589.37	‐	‐
R‐L2	3	23	‐	‐	3638.76 +/− 775.79	5938.69 +/− 290.09	‐	‐
R‐L2	4	10	NON	10	‐	‐	1135.13 +/− 242.01	996.21 +/− 143.45
R‐L2	5	11	GP	10	678.33 +/− 144.62	687.86 +/− 103.95	700.31 +/− 149.31	721.29 +/− 122.16
T‐M70	1	5	‐	‐	‐	‐	‐	‐
T‐M70	2	16	GP	15	628.68 +/− 134.03	1946.89 +/− 187.71	562.65 +/− 119.96	792.43 +/− 89.86
T‐M70	3	3	‐	‐	‐	‐	‐	‐
G2a‐U8	1	9	GP	9	‐	‐	774.65 +/− 165.15	871.71 +/− 136.85
G2a‐U8	2	20	‐	‐	3140.2 +/− 669.49	6764.46 +/− 348.90	‐	‐
G2a‐U8	3	5	GP	5	‐	‐	‐	‐

*Note:* Calculations are performed for whole clusters (Date_All) and for common‐specific individuals (Date_common).

Abbreviations: Common, name of the Common, if any, to which the cluster is specific (GP, Grignano P; MAS, Massenzatica; NON, Nonantola; SAB, S. Agata B.); Date_all, time estimate for all cluster individuals; Date_Common, time estimate for Common‐specific individuals only; Ncommon, number of Common‐specific individuals; Ntot, total number of individuals.

These results suggest that each of these Common‐specific clusters may have spread from a common recent ancestor that could have been living around or after the time of separation between Commons and Controls. Accordingly, we estimated the ages of all the identified clusters whose size exceeds nine individuals, both using Common‐specific individuals only (when available and in sufficient number) and the whole clusters. Being aware that time estimates—and particularly STR‐based estimates—must be taken with caution, we interpret Common‐specific estimates as lower limits to the age of foundation/segregation of Commons, while whole‐cluster estimates provide an idea of the temporal depth of the “general” genetic background.

In general, our results (Table 2) revealed a good agreement between the two adopted methods (*SD* estimator and Batwing), especially as concerns Common‐specific clusters. In fact, Common‐specific dates seem to point to the central period of Middle Ages up to the Early Modern Age, the only exception being the Massenzatica‐Mesola Cluster (R‐L51, Cluster 2, COM), which instead points to the late Antiquity. In contrast, ages of whole clusters, particularly those that are not associated to Commons, range from 2000 to more than 5000 years ago.

### 3.5 Model comparison

We then compared three demographic models for interpreting the observed differences between Commons and Controls (Figure 2). According to the first model, such differences are explained solely by drift after the establishment of the Commons (Figure 2a). The second and the third models instead add an external contribute to the genetic make‐up of the Commons, considering respectively a 30% and 50% admixture component (Figure 2b). Results for each of the Common/Control pair are summarized in Table 3, where each panel reports the classification error obtained from the comparison of the set of models. Firstly, we compared the three considered demographic models in a single run (Table 3, upper panel), which resulted in high classification errors, possibly due to high similarity between 30% and 50% admixture models. Accordingly, we decided to separately compare the Drift‐only model with each of the two “Admix” models (30% and 50%; Table 3, lower panels), which in fact produced a considerable reduction of classification errors. The four sets of comparisons (i.e., one for each Common/Control pair) revealed two opposite trends, with the Eastern Commons of Grignano P. and Massenzatica showing signals of substantial introgression from external sources, whereas for the Central Commons of S. Agata B. and Nonantola the model with only drift was strongly favored. In particular, for Massenzatica the evidence in favor of admixture was subtler (posterior probabilities of 0.60 in favor of Admix‐30 model when considering all models and of 0.53 when comparing Admix‐30 with Drift only, while the comparison between Admix‐50 and Drift only was in favor of the latter with a posterior probability of 0.77), while for Grignano P. the support for the admixture model was stronger, with Admix‐30 and Admix‐50 models showing posterior probabilities of 0.60 and 0.59 when compared with Drift‐only. Importantly, all the considered models proved to be able to reproduce the observed variation, as shown by linear discriminant analysis (LDA; Figure S3).

**TABLE 3:** Model comparison and selection for each Common/Control pair

Common	Control	Model	Classification error	Votes	Posterior probability
*All models in a single run*					
Grignano P.	Grignano P.	Drift only	0.28	154	
		Drift +30% admix	0.62	165	
		**Drift + 50% admix**	**0.39**	**181**	**0.49**
Massenzatica	Mesola	Drift only	0.28	158	
		**Drift + 30% admix**	**0.62**	**279**	**0.60**
		Drift +50% admix	0.39	63	
S. Agata B.	Nonantola	**Drift only**	**0.28**	**308**	**0.69**
		Drift +30% admix	0.62	144	
		Drift +50% admix	0.38	48	
Nonantola	Nonantola	**Drift only**	**0.29**	**365**	**0.70**
		Drift +30% admix	0.62	124	
		Drift +50% admix	0.39	11	
*Drift only* vs. *Drift + 30% admix*					
Grignano P.	Grignano P.	Drift only	0.26	225	
		**Drift + 30% admix**	**0.29**	**275**	**0.60**
Massenzatica	Mesola	Drift only	0.26	200	
		**Drift + 30% admix**	**0.29**	**300**	**0.53**
S. Agata B.	Nonantola	**Drift only**	**0.26**	**350**	**0.69**
		Drift +30% admix	0.29	150	
Nonantola	Nonantola	**Drift only**	**0.26**	**396**	**0.78**
		Drift +30% admix	0.29	104	
*Drift only* vs. *Drift + 50% admix*					
Grignano P.	Grignano P.	Drift only	0.20	233	
		**Drift + 50% admix**	**0.20**	**267**	**0.59**
Massenzatica	Mesola	**Drift only**	**0.20**	**389**	**0.77**
		Drift +50% admix	0.20	111	
S. Agata B.	Nonantola	**Drift only**	**0.20**	**393**	**0.85**
		Drift +50% admix	0.20	107	
Nonantola	Nonantola	**Drift only**	**0.20**	**485**	**0.94**
		Drift +50% admix	0.20	15	

*Note:* Bold characters indicate the selected models.

### 3.6 Parameters estimation

When performing model selection, we tested demographies accounting for different proportions of admixture (0%, 30%, and 50%) from an external source of genetic variation. This was done in order to maximize the identifiability of the considered models, which correlates with the degree of differentiation among the tested demographic histories. However, the admixture parameter could actually vary in a wider range than that explored by the above tested models. Accordingly, we estimated admixture rate for all the considered Commons using ABC (Table 4, Figure S4). Consistently with the model selection analysis, posterior probabilities in Nonantola and S. Agata B. appear narrower and shifted toward lower values than those observed for Grignano P. and Massenzatica. Median admixture rates in the Eastern Commons (Grignano P. and Massenzatica) were around 30% (with mean value of 56%) in Grignano P. and 20% (with mean value of 41%) in Massenzatica. In Central ones (S. Agata B. and Nonantola) they were around 10% with slightly narrower confidence intervals. In other words, a small amount of admixture/introgression in Nonantola and S. Agata B., which showed support for the model with “only drift,” is still compatible with the observed data.

**TABLE 4:** ABC estimator results describing admixture estimates between a non‐Italian ghost populations (Pop 2; see Figure 2) and each Common, showing mean, mode, median, and 95% highest posterior density (HPD) interval

Common/control	Mean	Mode	Median	95% lower HPD	95% upper HPD
**Grignano P./Grignano P.**	0.56	0.38	0.29	0.05	0.49
**Massenzatica/Mesola**	0.41	0.12	0.19	4 × 10^−5^	0.42
**S. Agata B./Nonantola**	0.27	0.05	0.11	2 × 10^−5^	0.34
**Nonantola/Nonantola**	0.21	0.04	0.08	3 × 10^−5^	0.26

## 4 DISCUSSION

The main aim of this study is to understand how admixture and drift may contribute to the genetic make‐up of peculiar populations such as those associated to the presence of Commons. At the same time, these groups, investigated at a micro‐geographic scale after a careful sampling, may reveal aspects of the recent genetic history of the region in which they are located—aspects that are normally hidden or more difficult to detect in the “general” population. Similar considerations were also expressed in a study about four Anatolian villages by Gokcumen et al. (2011), according to whom “broad, ethnicity‐based sampling is inadequate to capture the genetic signatures of recent social and historical dynamics, which have had a profound influence on contemporary genetic and cultural regional diversity”.

Indeed, a previous study about the *Partecipanza* of S. Giovanni in Persiceto (Boattini et al., 2015) suggested that social‐cultural features such as the presence and the persistence through the centuries of Commons seem particularly apt to produce, as a biological effect, peculiar micro‐geographic genetic structures in which both admixture and drift could have played an important role. Accordingly, we sampled four Commons from the Padana Plain, Northern Italy, each of them characterized by its peculiar set of rules to keep the property of the shared goods in the hands of a restricted and well‐defined group of stakeholders (Figure 1, Table 1). Then, we compared their Y‐chromosomal structure to that of Control populations, that is, groups that share the same environmental and cultural features of the Common, excepted for the affiliation to the Common itself.

Our results showed that in all cases Commons are characterized by a significant reduction of genetic diversity, compared with their Controls (Figure 3, Table S3). This was somehow anticipated, since the social–cultural rules determining the affiliation to a given Common are likely to have caused a certain degree of isolation between the Common and the neighboring populations, at least from the paternal side. Such rules are particularly stringent for Nonantola, S. Agata B. and Grignano P., where both local residence and legitimate patrilineal descent are required, while in Massenzatica only the former is applied (Table 1). Accordingly, only a moderate diversity reduction was observed in Massenzatica, while such phenomenon is more evident in the three remaining Commons, in addition showing a clear association with population size. Furthermore, as revealed by AMOVA, Control groups show an overall higher similarity between them than Commons do. A significant reduction of Y‐chromosomal diversity was not detected in the *Partecipanza* of S. Giovanni in Persiceto, but this fact could be due to the lower number of Y‐STRs and Y‐SNPs typed in that study (Boattini et al., 2015).

Differences between Commons and their Controls are also apparent when inspecting their haplogroup composition (Table S4). In fact, in all cases we detected an overall significant difference in terms of Fst between the considered pairs of populations. In particular, few haplogroups clearly characterize each of the considered populations. On that respect, a subtle difference between Central (Nonantola, S. Agata B.) and Eastern (Grignano P., Massenzatica) Commons emerged: Central Commons harbor haplogroups/paragroups that are among the most frequent in Northern Italy, i.e. R‐U152* (S. Agata B.) and R‐L2 (Nonantola); instead Eastern Commons show high frequencies of more uncommon or rare haplogroups/paragroups in Italy such as R‐L51* and T‐M70 (Boattini et al., 2013). All these cases can be explained based on the effects of genetic drift, according to which the frequency of some haplogroups may have increased (or decreased) by random fluctuations. However, it is possible that some of them—particularly in Eastern Commons—could be the result of an introgression/admixture event around the time in which the Common was formed. The same observations hold also for MDS results (Figure 3), in which the four Commons, compared with reference Italian and European populations, tend to occupy peripheral or even outlier positions in the genetic space: indeed, both drift and admixture could explain such configuration. In addition, the case of the *Partecipanza* of S. Giovanni in Persiceto—where similar patterns where observed, most notably the higher‐than‐expected frequency of the otherwise rare haplogroup I1‐L22—suggested that external admixture could have played a significant role in the genetic history of Commons (Boattini et al., 2015).

However, it is important to underline that isolation in the considered populations is here only examined from the Y‐chromosomal (patrilineal) point of view. Indeed, estimates of social endogamy in Grignano P. is ~60% and in S. Agata B. only ~30%, suggesting that marriages did not occur based on affiliation to the Common. In other words, Commons appear as “open” to the rest of the population from the maternal side. This fact implies that, differently from “classic” isolates, such as ethno‐linguistic minorities as the Arbereshe of Southern Italy or German‐speaking groups from Northern Italy (Anagnostou et al., 2017; Anagnostou et al., 2019; Boattini et al., 2011; Coia et al., 2012; Coia et al., 2013; Colonna et al., 2013; Esko et al., 2013; Sarno et al., 2016), no inbreeding increase is expected in these populations, nor significant genetic differentiation, if autosomal variants or mitochondrial DNA (mtDNA) would have been considered. Indeed, no significant difference was observed in S. Giovanni in Persiceto between the Common and its Control from the mtDNA point of view (Boattini et al., 2015).

We then explicitly tested the admixture‐drift model in our populations via ABC. A similar approach was recently used at a larger geographic scale in Kutanan et al. (2019), which however is here aimed at disentangling specific micro‐geographic patterns and reconstructing recent demographic histories. In particular, we considered three different models namely: (a) Drift‐only, (b) Admix‐30 (i.e., 30% admixture followed by drift), and (c) Admix‐50 (i.e., 50% admixture followed by drift) (Figure 2). Our simulations suggested that the Drift‐only model is favored for Central Commons (Nonantola, S. Agata B), while for Eastern Commons (Grignano P., Massenzatica) some degree of introgression from an external source of genetic variation is more probable (Table 3, Figure S3). However, admixture cannot be excluded also for Central Commons, albeit with a much lower admixture rate, as suggested by ABC parameter estimation (Figure S4). In fact, after estimating the admixture parameter, we obtained values around 17%–21% with large confidence intervals for Eastern Commons, and 11%–15% with tight confidence intervals for Central ones (Table 4). Interestingly, these results agree with the haplogroup dissection of the considered populations, in which Eastern Commons are characterized by a remarkable presence of infrequent haplogroups in Northern Italy.

As for the observed heterogeneity among the considered Commons, it should be mentioned here that similar results were found in different contexts, suggesting that dynamics of drift/admixture alike those described here may be at work also in these cases. For instance, Chaix et al. (2007) observed that in Central Asia lifestyle differences were associated to a substantial loss of Y‐chromosome diversity in pastoral populations compared with farmer ones. Similarly, Gokcumen et al. (2011) in central Turkey observed micro‐geographic patterns of paternal genetic structuring mostly associated to cultural isolation (ancestry, religion). Interestingly, both these examples revealed that such differences/structures were Y‐chromosome‐specific, being absent when considering mitochondrial DNA or autosomal DNA.

Our second aim was to use this information in order to reconstruct the genetic history of these groups in the wider context of Italian genetic history. Accordingly, we proceeded to a more detailed analysis of the detected haplotypes/haplogroups/paragroups using DAPC (Table 2, Table S5, Figure S2). Our results revealed 11 Common‐specific clusters of haplotypes within the most frequent haplogroups. The great majority (9/11) of them were exclusive or almost exclusive of one of the considered Commons, albeit with two exceptions, namely Cluster 3 in paragroup R‐U152*, which is associated to the Nonantola Common but is sporadically observed in other Commons and Controls, and Cluster 2 in hg R‐L51*, which is associated to the Common of Massenzatica, but is sporadically observed in all Controls and also in three central‐northern European populations (Bavarian, Danish, English). Interestingly, Massenzatica and Nonantola are also the Commons that showed the lowest amount of diversity reduction, suggesting a lower degree of paternal isolation than the other ones (Figure 3, Table S3).

In general, it seems plausible that Common‐specific clusters would mark expansion events within the corresponding populations, therefore estimates of their time depth should work as a lower bound for the time of origin of the Common itself.

A key parameter, when evaluating time estimates based on molecular markers, is the average generation time. This study suggests that, based on documented pedigrees, a generation time of 33.72 years could be adequate at least for Northern Italian populations. Indeed, our estimate overlaps with similar pedigree‐based estimations for the Common of S. Giovanni in Persiceto (33.38 years; Boattini et al., 2015) and, even more interestingly, for Emilia‐Romagna populations not including Commons (33.57 years; Boattini et al., 2019). These values substantially agree with previous estimates based on demographic cross‐cultural comparisons (Fenner, 2005), according to which male generation interval in “developed nations” is 30.8, and in “less developed nations” is 31.8. Such figures, which are slightly lower than ours, are referred to 20th centuries populations, while pedigree‐based estimates encompassing larger time intervals vary between 31.9 (Iceland, 1742–2002; Helgason et al., 2003) and 34.5 (French Canada, 1850–1990s; Tremblay & Vézina, 2000). Interestingly, our results hint at a moderate but significant local variability, with Grignano P. showing slightly higher values than S. Agata B.

Despite considering the fact that STR‐based estimates should be taken with the greatest caution, our SD and Batwing time estimates, besides being in agreement with each other, seem to be coherent with historical information about the origin of these communities (Arioti et al., 1990; Cori, 2011; Costato, 1968; Fregni, 1992; Venturoli, 2004). Indeed, dates (Table 2) range between central Middle Ages and early Modern Age, which is in agreement with an origin of the Commons at least 1000 years ago and following re‐expansion events after the segregation of the founder families (in Nonantola, S. Agata B., Grignano P.) around 500 years ago. Again, Massenzatica is an exception pointing towards an earlier origin—likely in Late Antiquity—which seems coherent with local archaeological remains (Cori, 2011). Whole clusters age estimates instead point especially to the interval between 2000 and 5000 years before present, which is in agreement with the more general make‐up of the Italian population (Antonio, 2019; Boattini et al., 2013; Cocca, 2020; Fernandes et al., 2020; Marcus et al., 2020; Sarno et al., 2017; Sazzini et al., 2016; Sazzini et al., 2020).

Recent studies based on modern and ancient genomes suggested that the “core” of Italian genetic variation was already in place in the early antiquity. Late antiquity and medieval migrations, as far as we are concerned, seem to have left only minor traces in the Italian genomic background, which are not apparent in “general” populations (Antonio, 2019). For instance, traces of late medieval migrations from Southern Balkans to Southern Italy are detectable in some ethnic‐linguistic minorities that still conserve their original language (Arbereshe) but not in the “average” Southern Italian population (Sarno et al., 2016; Sarno et al., 2017). Similarly, the *Partecipanza* of S. Giovanni in Persiceto was hypothesized to spot traces of an early medieval migration from Northern Europe which were lost in other groups (Boattini et al., 2015).

As above discussed, our analyses suggested that Eastern Commons, that is, Grignano P. and Massenzatica, are the most likely cases for admixture‐drift, while the Y‐chromosomal variability of Central Commons (Nonantola and S. Agata B.) is more easily explained by drift only. In light of these results together with DAPC and dating experiments, the most likely scenario could be the following. The three Commons with the patrilineal descent rule (Nonantola, S. Agata B., and Grignano P.) were founded in the central Middle Ages, while Massenzatica could have been a few centuries older. All communities likely stem from local populations that lived in the area from a long time, however Grignano P. and Massenzatica probably incorporated a ~ 20% contribute from an external population around the same time in which the Common was founded. Unfortunately, it is not possible to be more precise about the origin/identity of these external populations, limiting ourselves to the observation that Grignano P. exhibits an otherwise rare haplogroup, T‐M70, which could refer to a Mediterranean background, while R‐L51*, which is typical of Massenzatica, is mostly observed in central Europe (Busby et al., 2012; Harney et al., 2018; Mendez et al., 2011; Myres et al., 2011).

Later, isolation and genetic drift were induced in Nonantola, S. Agata B., and Grignano P. in virtue of their patrilineal separation from the neighboring populations. In Massenzatica, instead, we believe that the peculiar environment in which the community was founded—a lagoon‐like environment subjected to frequent floods from the Po river—played a fundamental role in its segregation.

## 5 CONCLUSION

This study shows how the co‐presence of admixture and drift forms a suitable model for explaining the genetic variability of at least two of the four considered Commons, namely Grignano P. and Massenzatica. At the same time, we observed that the peculiar social‐cultural features of Commons—based on patrilineal descent and local residence—influence their Y‐chromosomal variability in a way reminiscent of ethnic‐linguistic minorities, where phenomena such as isolation and/or admixture are frequently observed. The collected results allowed to reconstruct some aspects of the genetic history of the considered communities. For instance, our estimates suggest that the Commons of Nonantola, S. Agata B., and Grignano P. probably originated in the central Middle Ages from a set of mainly but not exclusively local populations, while the case of Massenzatica seems to suggest a more ancient origin. The same admixture‐drift model could be proposed as a reference model for the interpretation of the genetic structure of isolated populations in which social‐cultural features play a significant role.

## AUTHOR CONTRIBUTIONS

**Stefania Sarno:** Data curation; formal analysis; methodology; supervision; visualization; writing‐original draft. **Rajiv Boscolo Agostini:** Formal analysis; methodology; writing‐original draft. **Sara De Fanti:** Formal analysis; methodology. **Gianmarco Ferri:** Formal analysis; methodology. **Silvia Ghirotto:** Formal analysis; methodology; writing‐original draft. **Giorgia Modenini:** Data curation; investigation. **Davide Pettener:** Conceptualization; resources; supervision. **Alessio Boattini:** Conceptualization; data curation; formal analysis; investigation; methodology; supervision; writing‐original draft.

## CONFLICT OF INTEREST

The authors declare no conflict of interest.

## Supporting information

**Supplementary Figure 1** Schematic representation of paternal pedigrees for S. Agata B. (1‐24) and Grignano P. (25–61) samples. Numbers along each branch represent the corresponding number of generations. Red branches point to individuals which were excluded from genotyping given their recent relatedness with other individuals.

**Supplementary Figure 2** DAPC of Y‐STR variation in haplotypes from Commons, Controls and comparison reference populations for the considered haplogroups (R1b‐U152, R1b‐L51, R1b‐L2, G2a‐U8, T‐M70). Scatterplot of the first and the second discriminant functions are reported.

**Supplementary Figure 3** LDA plots of the model comparison for each Common/Control pair. The observed data is represented as a straight line. 1) Grignano P. 2) Massenzatica/Mesola 3) S. Agata B./Nonantola 4) Nonantola. a) Drift only model (yellow) vs Drift +30% admix model (blue), b) Drift only model (yellow) vs. Drift +50% admix model (blue).

**Supplementary Figure 4** Posterior probability for the admixture rates between an external source of variation and the four Commons analyzed. a) Grignano P. b) Massenzatica c) S. Agata B. d) Nonantola.

**Supplementary Table 1** Full description of the model parameters used in Approximate Bayesian Computation (ABC).

**Supplementary Table 2** Parameters used for estimating average generation time and social endogamy in the 61 reconstructed pedigrees. Nind: number of individuals; NgenTOT: total number of generations; NgenAGE: number of generations with age information; NgenAGE1800: number of generations with age information after year 1800 (Grignano P. only); NRyearsTOT: total number of encompassed years; NRyears1800: total number of encompassed years after 1800 (Grignano P. only). The total number of marriages included within each genealogy (Ntotal) is subdivided in endogamic, esogamic and NA (i.e. incomplete due to missingness of bride's surname). Bold figures include additional generations/marriages from collateral branches not included in Supplementary Figure 1.

**Supplementary Table 3** Y‐chromosome diversity indexes based on STR haplotypes and haplogroup frequencies data.

**Supplementary Table 4** Y‐haplogroup frequencies in the considered Commons, Controls and European Comparison Populations.

**Supplementary Table 5** Absolute frequencies of DAPC Clusters in Commons, Controls and reference populations. Italic figures indicate Common‐specific Clusters.

## Data Availability

The data that support the findings of this study are openly available in YHRD at yhrd.org, reference numbers YA004724 and YA004725.
