# Supplementary material for: Y‐chromosome variability and genetic history of Commons from Northern Italy
Source: Am J Phys Anthropol. 2021 May 10;175(3):665–79. doi: 10.1002/ajpa.24302 (PMC8360088; doi:10.1002/ajpa.24302)

Supplementary Figure 1. Schematic representation of paternal pedigrees for S. Agata B. (1-24) and Grignano P. (25-61) samples. Numbers along each branch represent the corresponding number of generations. Red branches point to individuals which were excluded from genotyping given their recent relatedness with other individuals.

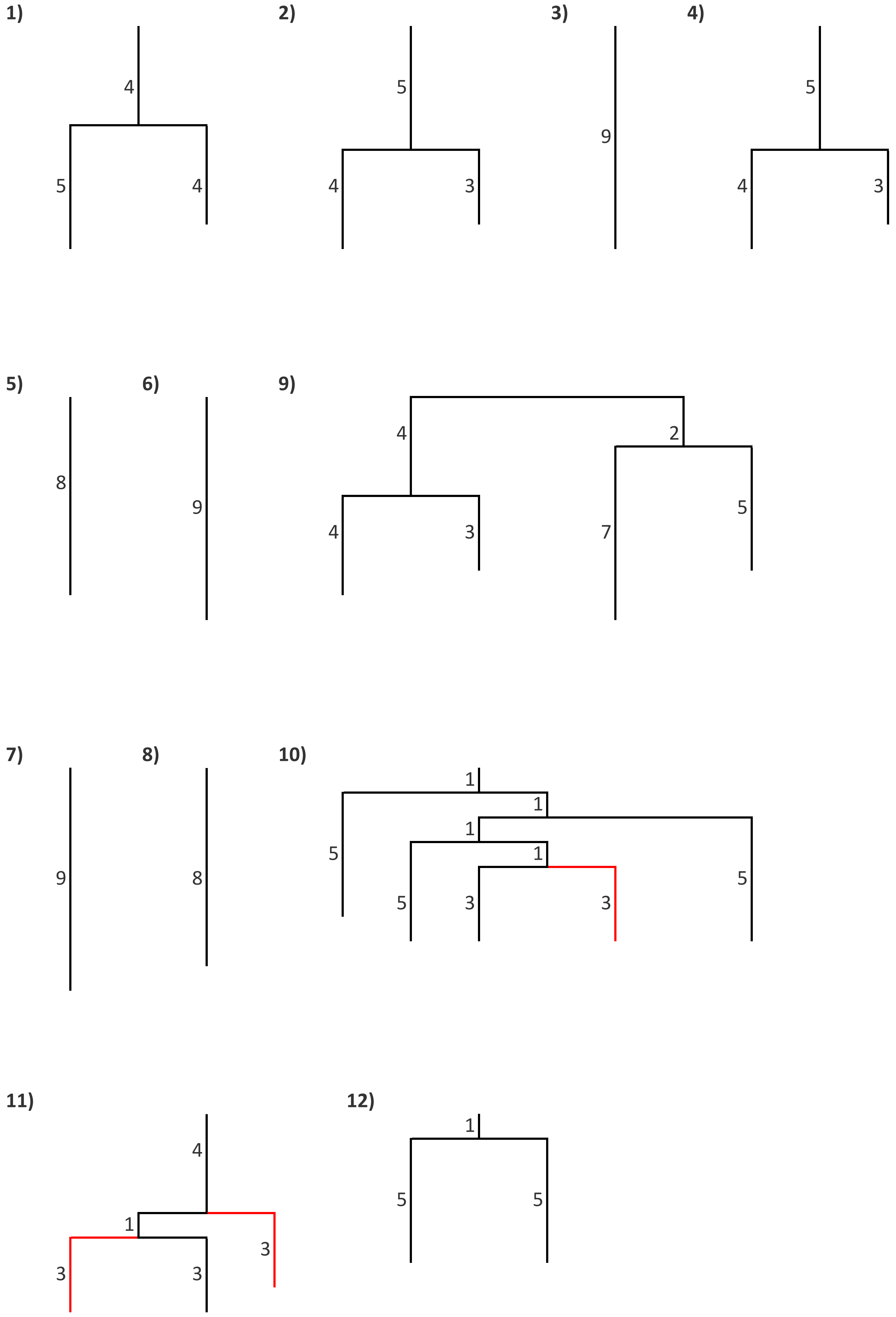

13)

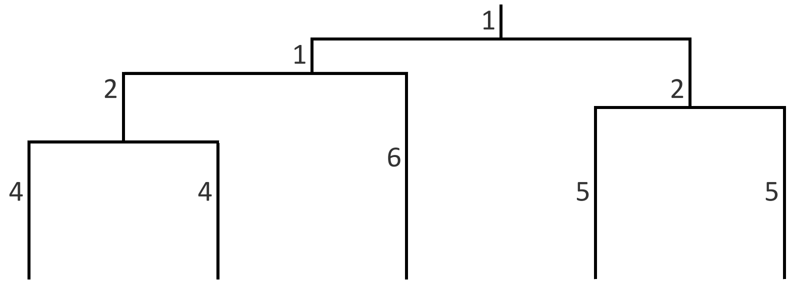

14)

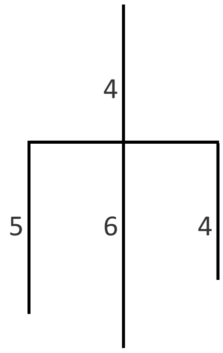

15)

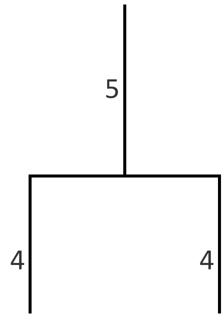

16)

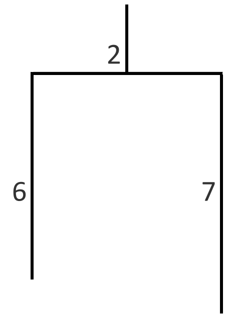

17)

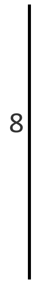

18)

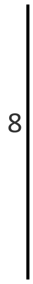

19)

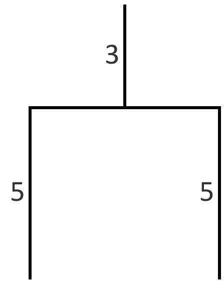

20)

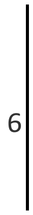

21)

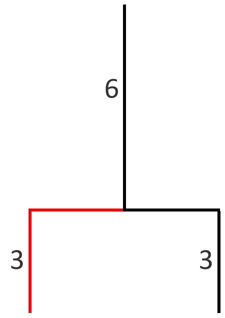

22)

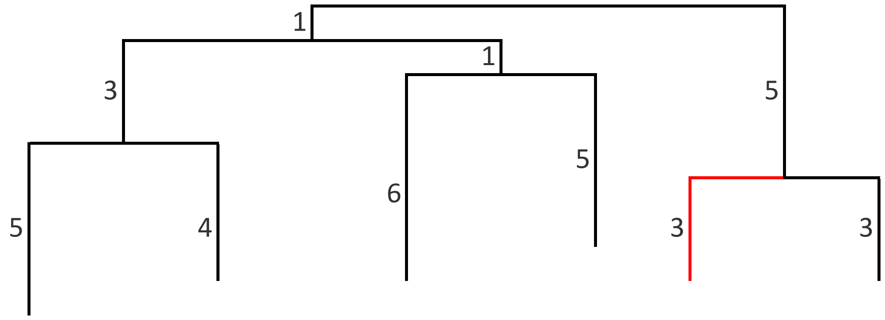

23)

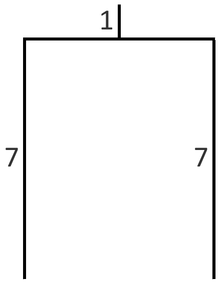

24)

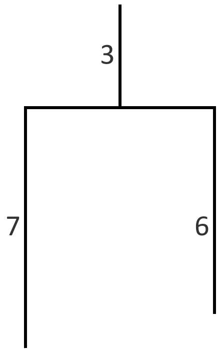

25)

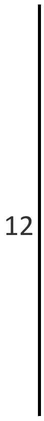

26)

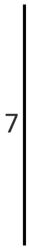

27)

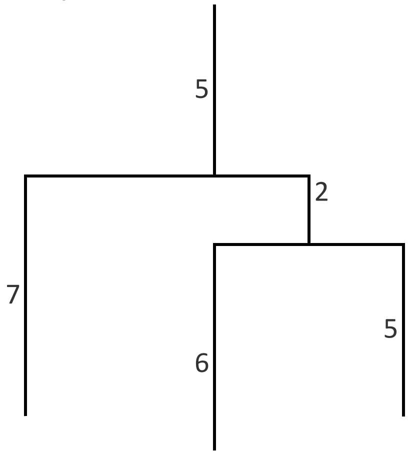

28)

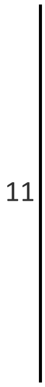

29)

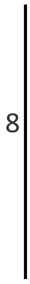

30)

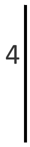

31)

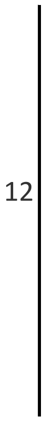

32)

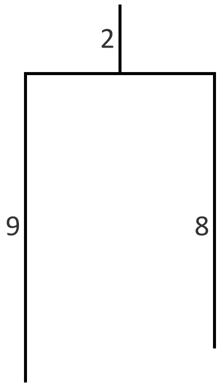

33)

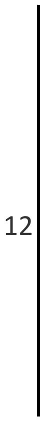

34)

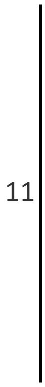

35)

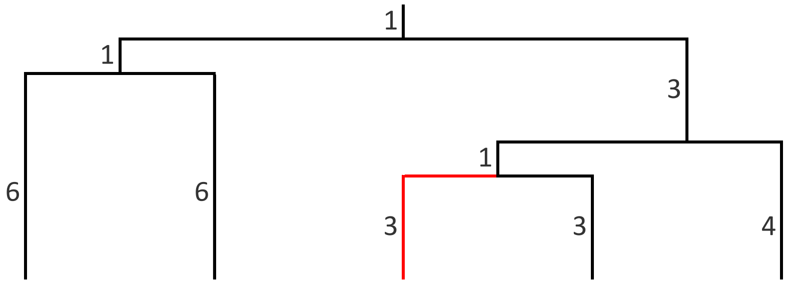

36)

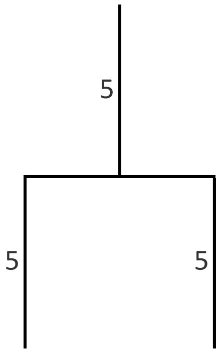

37)

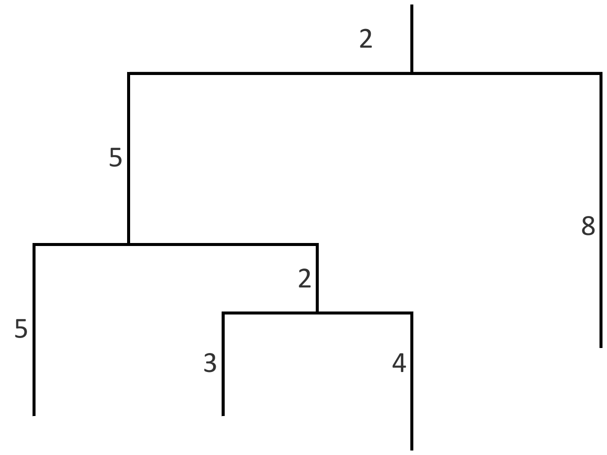

38)

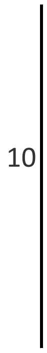

39)

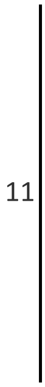

40)

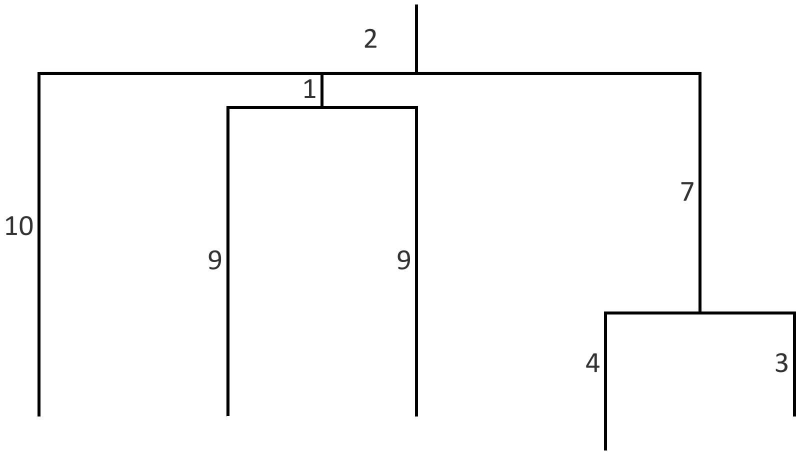

41)

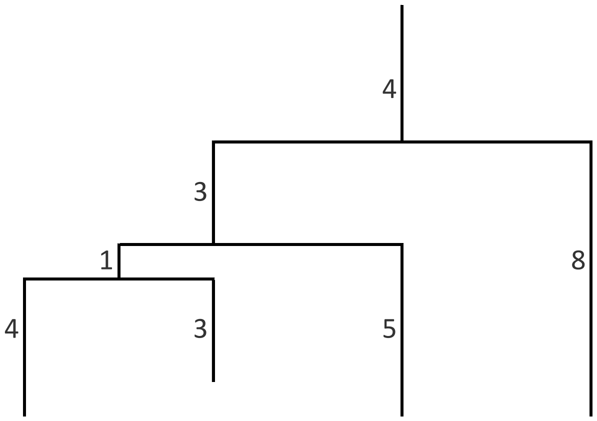

42)

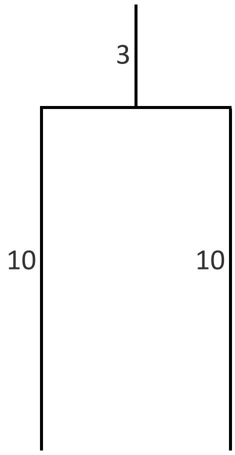

43)

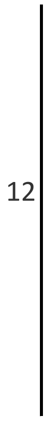

44)

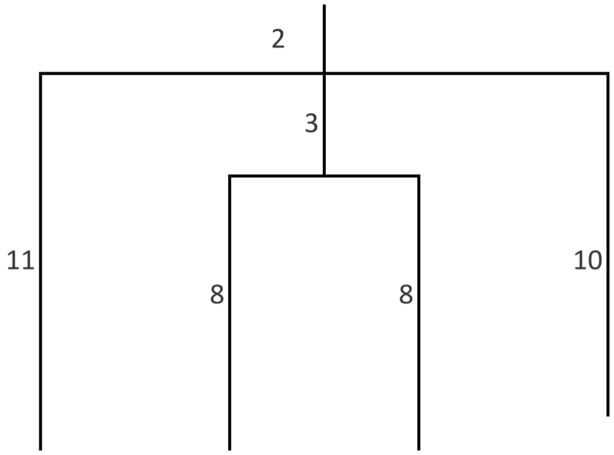

45)

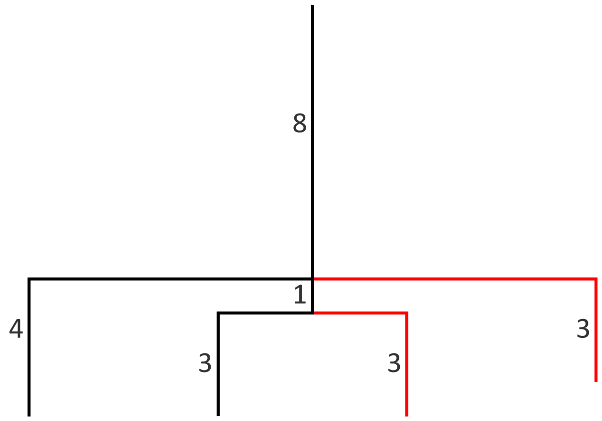

46)

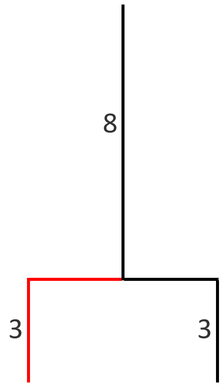

47)

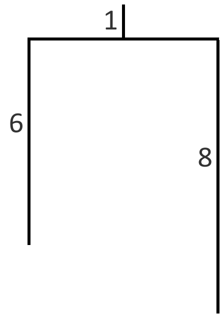

48)

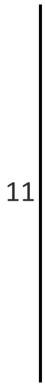

49)

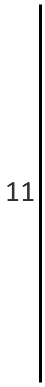

50)

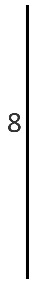

51)

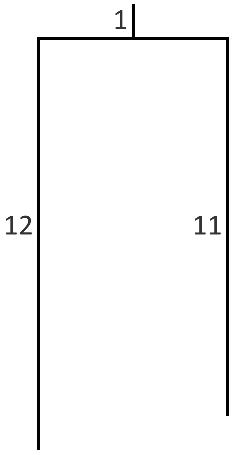

52)

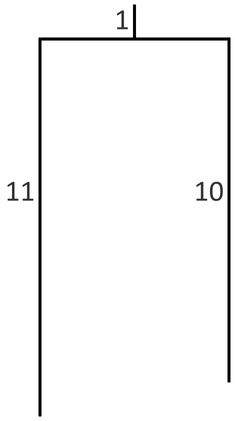

53)

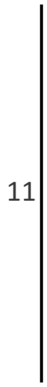

54)

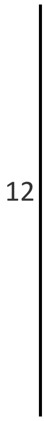

55)

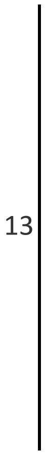

56)

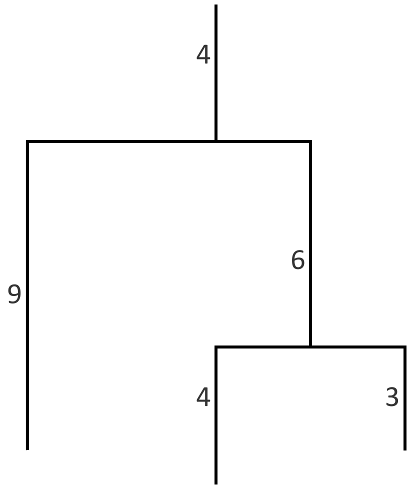

57)

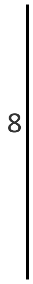

58)

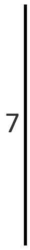

59)

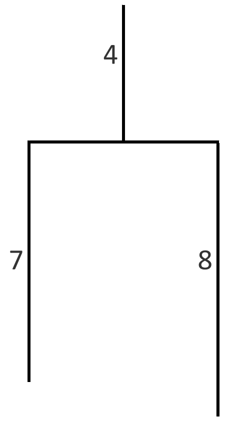

60)

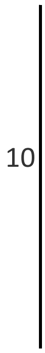

61)

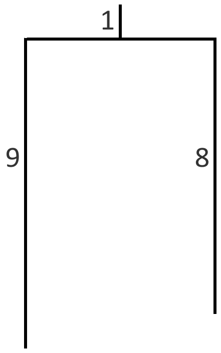

Supplement: Supplementary file 1 — Supplementary Figure 1 Schematic representation of paternal pedigrees for S. Agata B. (1‐24) and Grignano P. (25–61) samples. Numbers along each branch represent the corresponding number of generations. Red branches point to individuals which were excluded from genotyping given their recent relatedness with other individuals. [file AJPA-175-665-s002.pdf]
