# Supplementary figures and images for: Y‐chromosome variability and genetic history of Commons from Northern Italy
Source: Am J Phys Anthropol. 2021 May 10;175(3):665–79. doi: 10.1002/ajpa.24302 (PMC8360088; doi:10.1002/ajpa.24302)

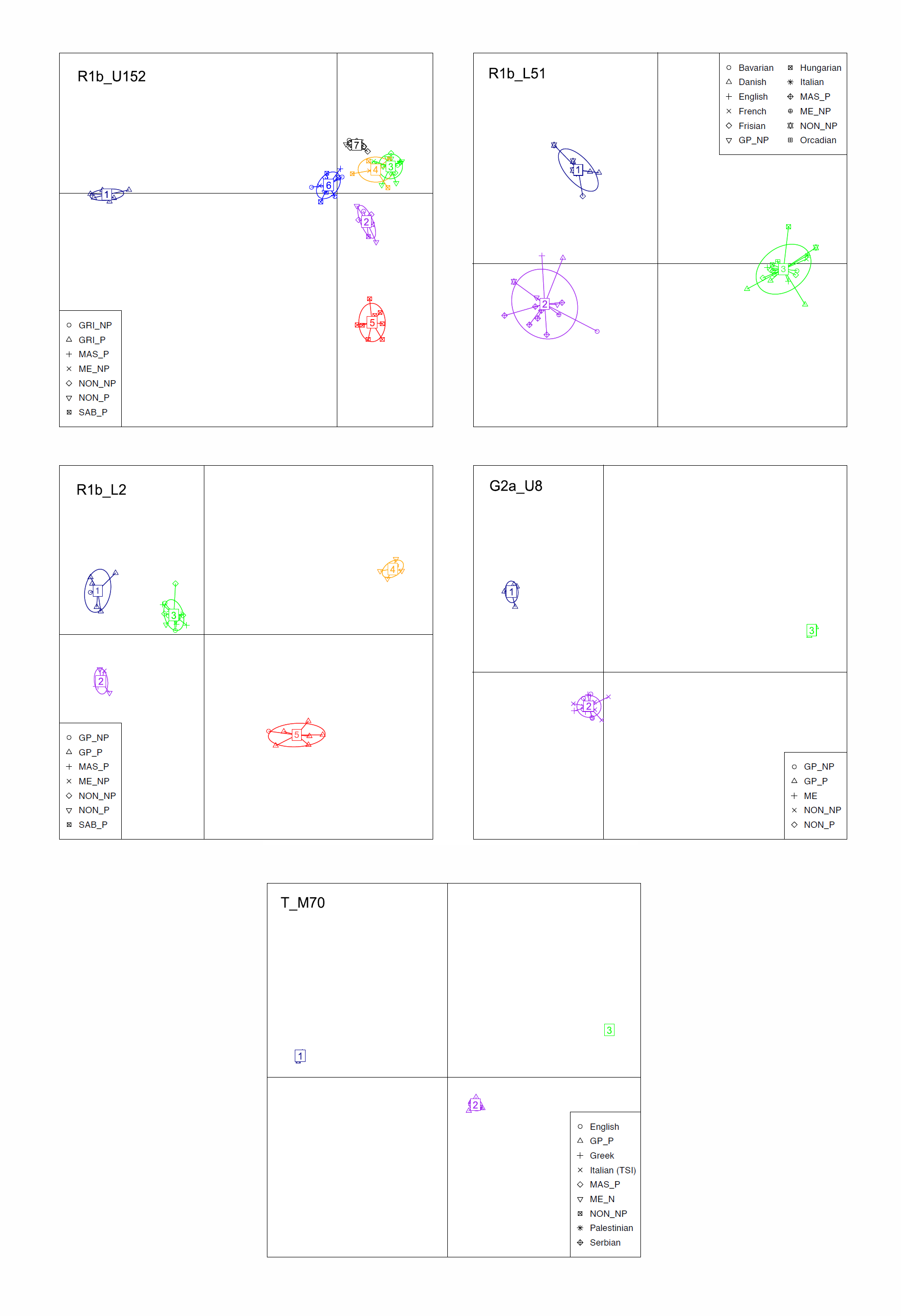

Supplement: Supplementary file 2 — Supplementary Figure 2 DAPC of Y‐STR variation in haplotypes from Commons, Controls and comparison reference populations for the considered haplogroups (R1b‐U152, R1b‐L51, R1b‐L2, G2a‐U8, T‐M70). Scatterplot of the first and the second discriminant functions are reported. [file AJPA-175-665-s003.tif]

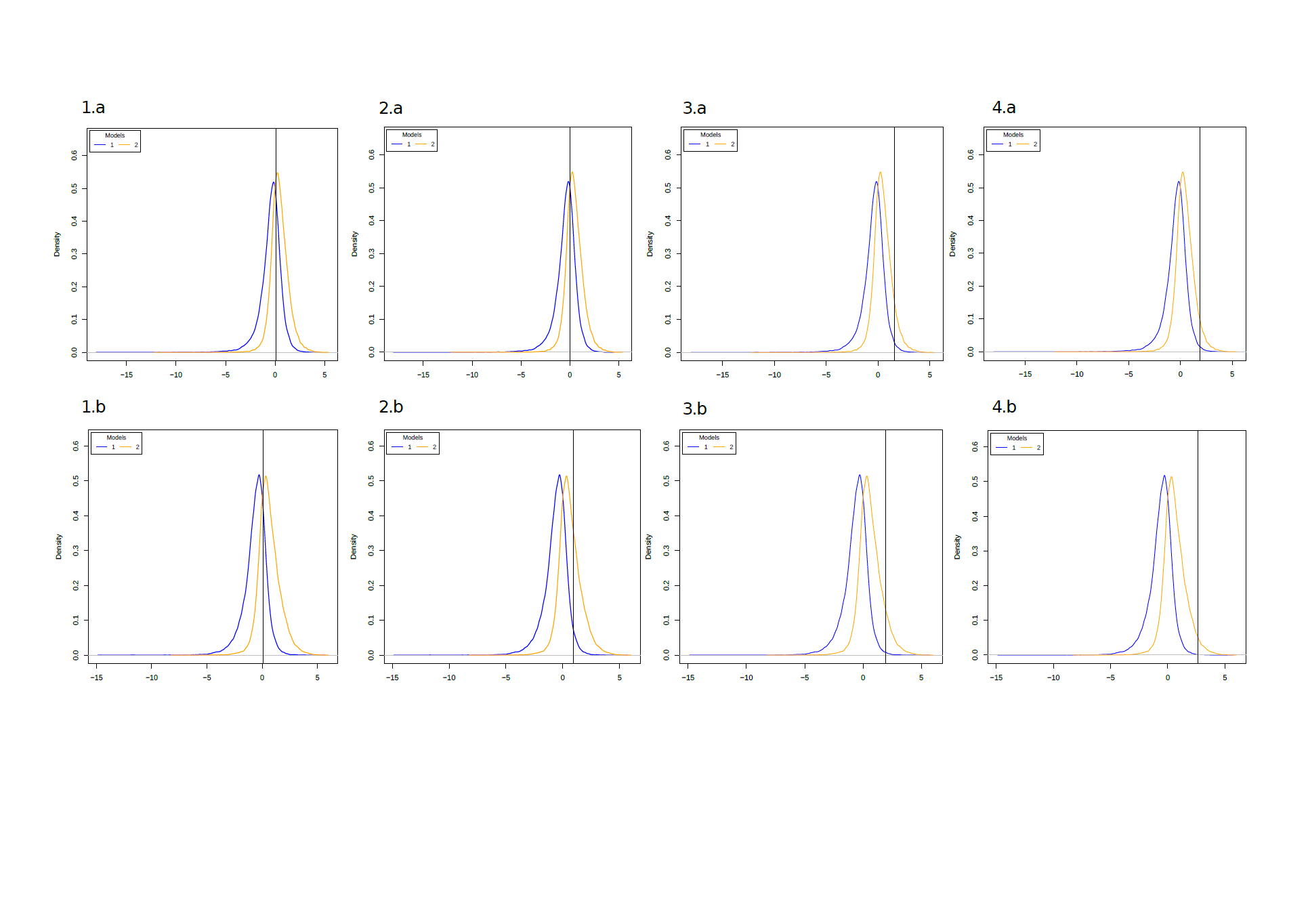

Supplement: Supplementary file 3 — Supplementary Figure 3 LDA plots of the model comparison for each Common/Control pair. The observed data is represented as a straight line. 1) Grignano P. 2) Massenzatica/Mesola 3) S. Agata B./Nonantola 4) Nonantola. a) Drift only model (yellow) vs Drift +30% admix model (blue), b) Drift only model (yellow) vs. Drift +50% admix model (blue). [file AJPA-175-665-s001.tif]

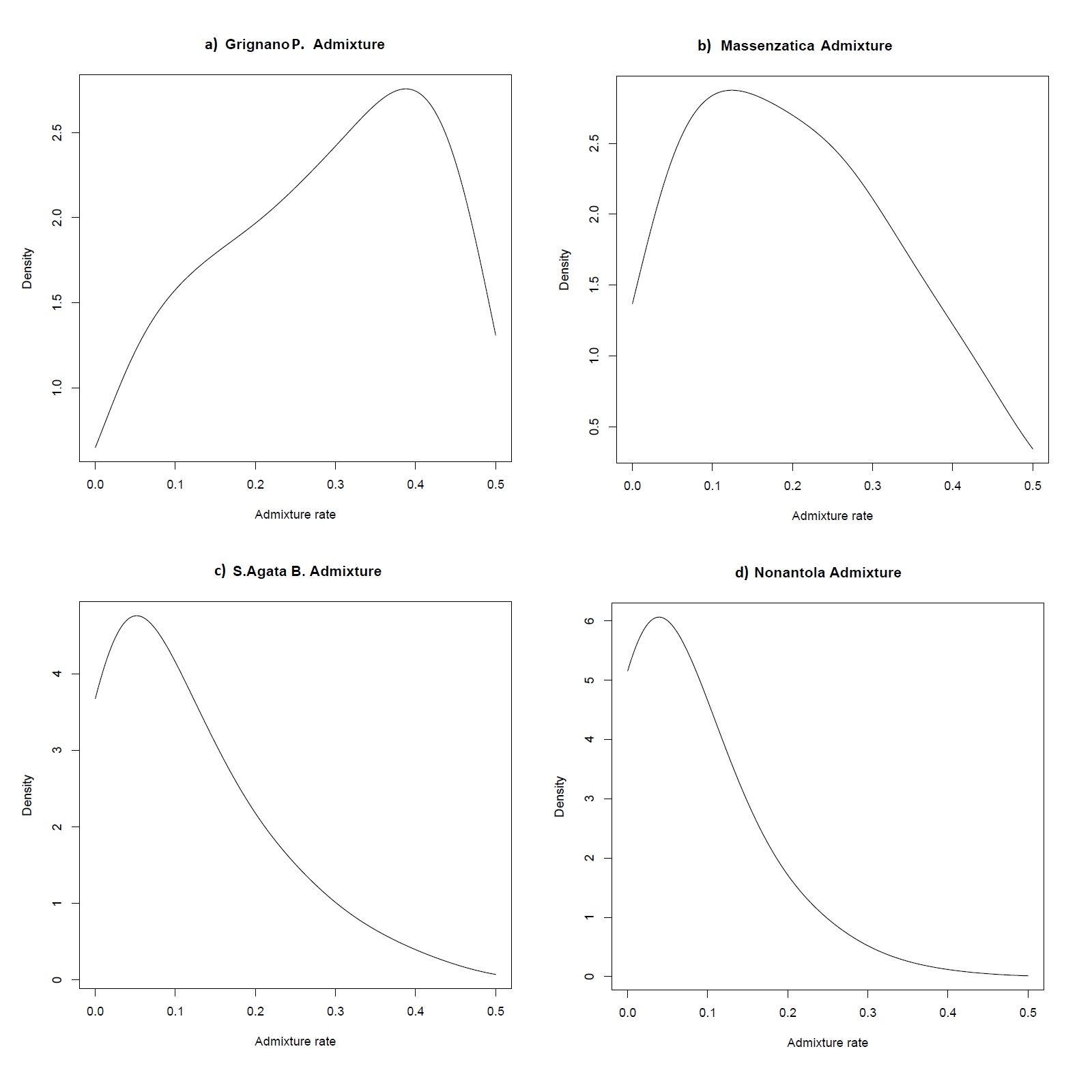

Supplement: Supplementary file 4 — Supplementary Figure 4 Posterior probability for the admixture rates between an external source of variation and the four Commons analyzed. a) Grignano P. b) Massenzatica c) S. Agata B. d) Nonantola. [file AJPA-175-665-s008.tif]
